# Defining the Benefit Package of Thailand Universal Coverage Scheme: From Pragmatism to Sophistication

**DOI:** 10.15171/ijhpm.2019.96

**Published:** 2019-11-02

**Authors:** Viroj Tangcharoensathien, Walaiporn Patcharanarumol, Waraporn Suwanwela, Somruethai Supangul, Warisa Panichkriangkrai, Hathairat Kosiyaporn, Woranan Witthayapipopsakul

**Affiliations:** ^1^International Health Policy Program, Ministry of Public Health, Nonthaburi, Thailand.; ^2^National Health Security Office, Bangkok, Thailand.

**Keywords:** Health Benefit Package, Health Insurance, Essential Medicines List, Universal Health Coverage, Thailand

## Abstract

Benefit package is crucial for implementing universal health coverage (UHC). This editorial analyses how the benefit package of the Thai Universal Coverage Scheme (UC Scheme) evolved from an implicit comprehensive package which covered all conditions and interventions (with a few exceptions), to additional explicit positive lists. In 2002 when the Thai UC Scheme was launched; the comprehensive benefit package, including medicines in the national essential list of medicines, formerly offered by the previous schemes were pragmatically adopted. Later, when capacities of producing evidence on health technology assessment (HTA) increased, rigorous assessment of cost effectiveness is mandatorily required for inclusion of new interventions into the Thai UC Scheme benefit package. This contributed to evidence-informed policy decisions. To prevent emptied promises, whichever policy choices are made about the benefit package, either using a negative or a positive list, developing country governments need to make quality health services available and accessible by the entire population. Political decision on benefit package should be informed by evidence on cost effectiveness, equity dimension and health system capacity to deliver equitable services. Low- and middle-income countries need to strengthen HTA capacity to generate evidence and inform policies.

## Background

Decision-making about the benefit package is a key entry point in implementing universal health coverage (UHC). Several prioritizing criteria are used such as burden of disease, cost-effectiveness of interventions, budget impact, equity^1^ and capacities of health systems to deliver. However, it is the political decisions, within the government fiscal capacity, at the end.^2,3^

Most OECD countries apply a positive list of medicines in their benefit package but there is no clear pattern of applying positive or negative lists for medical interventions.^4^ Thailand, however initially applied an implicit benefit package with a negative list but is now expanding the number of items in the positive list, ascertained through rigorous reviews using health technology assessments (HTAs) and other concerns.

Thai populations are covered by three public health insurance schemes since 2002. The civil servants and the pensioners, including their dependants (parents, spouses and children below 20 years old) are covered by the Civil Servant Medical Benefit Scheme (CSMBS). This scheme is financed solely by annual budget allocation and managed by Comptroller General’s Department of the Ministry of Finance. Private sector employees are mandatorily covered by Social Health Insurance (SHI) which is managed by Social Security Office, Ministry of Labour.^5^ The sources of finance come from equal contributions by employees, employers and the government.

The remaining populations are covered by the Universal Coverage Scheme (UC Scheme) which is financed by the government annual budget and managed by the National Health Security Office (NHSO). The latest national Health and Welfare Survey 2017 reported 99.2% population coverage by insurance schemes^6^; of which 75.7% by UC Scheme, 17.2% by SHI and 7.1% by CSMBS.

Given the comprehensive benefit package and minimum copayment at point of service by all three public insurance schemes, the data from World Development Indicators shows that the out-of-pocket payments by households had reduced from 34.2% of current health expenditure in 2000 to 12.1% in 2016.^7^ Domestic general government health expenditure increased from 13% to 15.3% of total government expenditure in the same period. This has resulted in low prevalence of catastrophic health spending and health impoverishment, a high level of UHC index, and a low level of unmet health needs.^8^ This is the result of increased government spending on health; and a comprehensive benefit package; with literally zero copayment.

These favourable achievements are gained despite the turbulent political climate and protracted political conflicts.^9^ Between 2001 and 2018, the UC Scheme thrived eight rival governments, six elections, two coup d’états and 13 health ministers.^10^ This reflects that UC Scheme is gradually owned by the people who had benefited from it; not by the political party who initiated it. Despite the decline in gross domestic products (GDP) growth (-0.7% in 2009), the UC Scheme budget had increased, reflecting sustained political and financial commitment across governments (see Figure). In addition, the extensive geographical coverage of primary healthcare and referral to secondary and tertiary hospital care are the core implementation platform, which results in favourable UHC outcomes.

**Figure:**
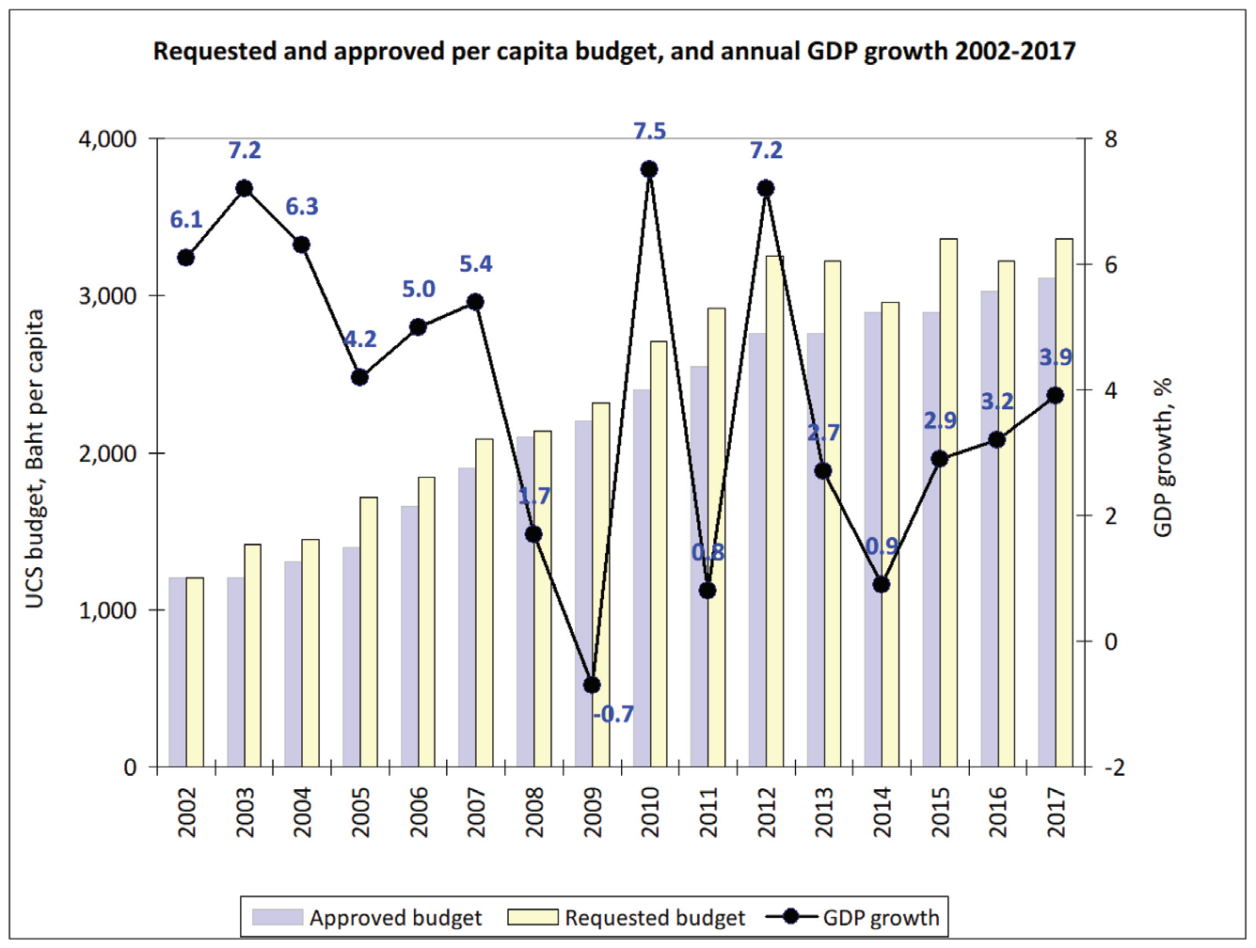
Requested and Approved Per Capita Budget of UC Scheme, and Annual GDP Growth, 2002-2017. Abbreviations: GDP, gross domestic products; US, Universal Coverage.
Source: Authors’ synthesis from NHSO annual reports on budget request and approval; and GDP growth from World Development Indicators.

This editorial analyzes and discusses how the benefit package applied by the UC Scheme evolved from an implicit package where all conditions and interventions were covered, except for a few in the negative list, to an application of and increased number in the positive list in recent years.

## Benefit Package of UC Scheme: Analysis of Historical Evolution

### Pragmatic Approach: Comprehensive Package With a Negative List

Prior to UHC in 2002, there were four insurance schemes covered 70% of population. These were: (1) the Medical Welfare Scheme for the low-income, the elderly, children under 12 years and disabled people; (2) the voluntary health insurance scheme for non-poor households; (3) the CSMBS; and (4) the SHI. Historically, the benefit package offered by these four schemes was comprehensive, covering outpatient and inpatient services, accidents and emergencies; all included medicines in the National List of Essential Medicines (NLEM). There were a few items in the negative list such as aesthetic surgery, treatment of infertility and transgender operations. Some health promotion interventions were provided by Ministry of Public Health.

At the 2002 reform, the UC Scheme combined the Medical Welfare Scheme, the voluntary insurance and included the 30% uninsured population. Given the path dependency, it continued to apply a comprehensive benefit package, with a few items in the negative list including antiretroviral treatment for HIV and treatment of end-stage renal failure due to high costs and health system inability to equitably deliver services. This comprehensive package also covered health promotion and disease prevention services for the whole population.

Analysis showed that two drivers influenced the adoption of a comprehensive package with a negative list for the UC Scheme. Firstly, UHC was a political manifesto at the January 2001 election, and needed to be achieved in a year. Thailand did not have the capacity for a thorough HTA and time did not allow for an extensive assessment about what interventions should be covered. At the 2002 reform, there were no discussions on what the basic minimum package should look like and what the costs of delivering these services should be.

Secondly, taking out non cost-effective services, previously provided by Medical Welfare Scheme and voluntary health insurance, was politically unpalatable and socially unacceptable. It took one year following the January 2001 election to achieve nationwide coverage by UC Scheme, with a pragmatic application of a negative list for health services.

### Pharmaceutical Benefit: Explicit Positive List of National Essential Medicines

The UC Scheme applies a positive list for pharmaceutical benefits, which covers all medicines in the NLEM. Medicines outside the NLEM are also covered if clinically indicated. The NLEM is subjected to regularly reviews based on health needs, safety, efficacy, cost-effectiveness, budget impact and affordability. The review requires all national experts in the 22 therapeutic groups to declare conflicts of interest to ensure transparency and objectivity.

The Health Economics Working Group, tasked by the NLEM sub-committee, generates evidences on cost effectiveness--with reference to a cost-effectiveness threshold of 160 000 Thai Baht (US$ 5333; exchange rate 30 Baht per US$) for one quality adjusted life year gained,^11^ budget impact, equity and delivery capacities, while the Price Negotiation Working Group negotiates for the lowest possible prices. When the price reaches the threshold, and if the budget impact is large, the price will be further negotiated. Thailand also applied government uses licenses to improve access to life saving medicines^12^ despite political pressure from the US government and European Commission to apply other mechanisms.^13^

In 2004, the NLEM moved from a minimum list to a reimbursement list of 692 active pharmaceutical ingredients. In 2008, the NLEM covered high-cost specialized medicines where patients could access them through an authorization system by specialists in the field. Anticancer, antiplatelet, drug effecting immune response and drug for genetic disorders, orphan medicines and antidotes were added to the NLEM in 2010^14^; A study shows it has improved access to these medicines.^15^ The current 2019 NLEM consists of 857 medicines. The number of items in the Thailand’s NLEM is close to that of World Health Organization (WHO) model list of essential medicines (see Table).

**Table:** NLEM 2019, Compared With WHO Model List of Essential Medicines, March 2017

**Group**	**Drug Group**	**Items in WHO Model List of Essential Medicines (2017)**	**Items in NLEM of Thailand (2019)**
1	Anaesthesia	14	9
2	Medicine for pain and palliative care	22	20
3	Antiallergics and medicines used in anaphylaxis	5	4
4	Antidotes and other substances used in poisonings	15	12
5	Anticonvulsants/antiepileptics	11	10
6	Anti-infective medicines	153	100
7	Antimigraine medicines	4	4
8	Antineoplastic and immunosuppressives	53	44
9	Antiparkinson medicines	2	1
10	Medicines effecting the blood	16	13
11	Blood products of human origin and plasma substitutes	12	12
12	Cardiovascular medicines	30	30
13	Dermatological medicines	18	13
14	Diagnostic agents	7	2
15	Disinfectants and antiseptics	7	7
16	Diuretics	5	5
17	Gastrointestinal medicines	11	11
18	Hormones, other endocrine medicines and contraceptives	31	22
19	Immunological	24	14
20	Muscle relaxants and cholinesterase inhibitor	6	5
21	Ophthalmological preparations	16	11
22	Oxytocin and antioxytotic	5	5
23	Peritoneal dialysis solution	1	1
24	Medicine for mental and behavioral disorders	17	15
25	Medicines acting on the respiratory tract	6	6
26	Solutions correcting water, electrolyte and acid-base disturbances	9	8
27	Vitamins and minerals	12	9
28	Ear, nose, and throat medicines	4	3
29	Specific medicines for neonatal care	6	4
30	Medicines for disease of joints	8	8

Abbreviations: NLEM, National List of Essential Medicines; WHO, World Health Organization.

### From Implicit to Explicit High-Cost Interventions

Recognizing challenges of access to certain high-cost interventions both within and outside the implicit benefit package, the Benefit Package sub-committee of the National Health Security Board (NHSB) gradually approved interventions (both services and medicines) and published them in the Royal Gazette and NHSB’s order as a positive list.^8^

High cost and new interventions were gradually added to the benefit package, for example, universal antiretroviral treatment (including voluntary counselling and testing, CD4 monitoring, viral load test and condom promotion) in 2006 and ‘detect and treat’ policy for any CD4 counts in 2016; renal replacement therapy using Peritoneal Dialysis First policy in 2009^16^; seasonal influenza vaccination for high risk groups in 2009; treatment of chronic kidney diseases to prevent clinical progression towards end-stage renal failure in 2011; liver transplantation for hepatic failure patients below 18 years old in 2011.

Inclusions of these interventions in the positive list of the UC Scheme’s benefit package are also subject to a rigorous assessment by several criteria including HTA, budget impact, equity, supply-side capacity and catastrophic impact to the patients or households. These complex multi-dimensional criteria for coverage decisions contributed to evidence-informed political decisions.^17^ Although renal replacement therapy was not cost-effective and had large budgetary impact, it was catastrophic to households and created inequity across the schemes, as CSMBS and SHI members were covered. The political decision taken through the Cabinet Resolution on universal renal replacement therapy for the UC Scheme in 2009 was based on ethical concerns, life saving measures and prevention of catastrophe.^16^

Importantly, the government approved additional budget through Cabinet Resolutions for new interventions which were not covered by the previous benefit package in order to ensure no “unfunded mandates.” If the NHSB singles out certain interventions already covered and funded in the comprehensive package paid by capitation for outpatient services, and Diagnostic Related Group for inpatient services, part of the existing budget is earmarked for these interventions to improve access.

## Discussion

The Thailand example of benefit package development provided a number of useful lessons to low- and middle- income countries.

First, there are two policy choices, either comprehensive package with the application of a negative list or application of a positive list. Countries applying a positive list should be cautious about itemized fee-for-service payments which can trigger supplier-induced demand and inefficiencies.^18^ The under-provision of services in the implicit comprehensive package can be minimized by clinical audits, and strong adherence to clinical practice guidelines.^19^ Singling out certain interventions in the implicit benefit package and apply additional incentive can improve access of interventions with limited access.

Whichever policy choices are made, governments need to make quality health services available to the entire population and subsidize the poor and vulnerable population to prevent financial barriers. Adequate funding to UC Scheme through full cost subsidies in the capitation payment for outpatient services, and Diagnostic Related Group payment for inpatient services prevents under-provision of services and is effective to prevent balance billing by healthcare providers.

Second, implicit benefit package with clear negative list is more practical as it takes shorter time to develop than an evidence-informed positive list provided that a political window opens briefly and might not stay open very long.^20^ Using HTA in producing a positive list can later fix the gaps of access to new interventions, but a secure new budget is required to prevent unfunded mandates.

Third, inclusion of new medicines and interventions to the benefit package should be subjected to a rigorous review. In addition to cost effectiveness, equity in access and health systems readiness to provide new interventions are equally important as decisions are not always simple.

For example, certain cost-effective interventions, such as dental root implants, are not covered as these services are available only in a few urban centres and that access can be inequitable.^8^ On the contrary, the non-cost–effective renal replacement with high budget impact was covered because self-payment for dialysis could bankrupt the households. Peritoneal Dialysis First policy in 2009 was adopted based on equity concern as traveling cost to hemodialysis centers in provincial hospital three times a week was prohibitive to rural patients. Instead, peritoneal dialysis could be managed at home with dialysis solution supplied to the patient’s home.^15^ Whichever decision is made, it must be fully informed by evidence. Low- and middle-income countries need to strengthen its HTA capacities.

Fourth, the inclusion of new medical products and technologies to the benefit package, despite being cost effectiveness, could lead to imbalance proportion of curative and disease prevention and health promotion expenditure such as in the case of Thailand during the last 15 years. The government should invest more in effective primary prevention which yields great health outcomes.^21^

## Conclusion

In 2002, as influenced by path dependence from previous health insurance schemes which offered comprehensive benefit package; and limited capacities to generate HTA evidence for prioritization of interventions for inclusion and exclusion from the benefit package, the UC Scheme applied a pragmatic approach using a negative list for health services to accommodate political commitment towards achieving full population coverage in a year.

Being aware of limited access to certain high-cost new interventions, the NHSO applied a more explicit positive list of new interventions with secured new budget or singled out some items from the existing benefit package with earmarked budget to boost access through additional incentives. Prioritizations of interventions are subject to rigorous HTAs of (*a* ) cost effectiveness, (*b* ) long term budget impact analysis, (*c* ) health systems capacity to deliver new interventions equitably, (*d* ) other ethical and equity considerations.

## Acknowledgement

Funding support was provided by Thailand Science Research and Innovation (TSRI) Contract No. RTA628000.

## Ethical issues

Not applicable.

## Competing interests

Authors declare that they have no competing interests.

## Authors’ contributions

VT designed the manuscript. All authors contributed to drafting and revising the manuscript, and gave final approval of the version to be published.

## Authors’ affiliations

^1^International Health Policy Program, Ministry of Public Health, Nonthaburi, Thailand. ^2^National Health Security Office, Bangkok, Thailand.
